# Experiences of Being a Parent to a Child with Amelogenesis Imperfecta

**DOI:** 10.3390/dj7010017

**Published:** 2019-02-09

**Authors:** Gunilla Pousette Lundgren, Tove Hasselblad, Anna Stigsdotter Johansson, Anna Johansson, Göran Dahllöf

**Affiliations:** 1 Division of Orthodontics and Pediatric Dentistry, Department of Dental Medicine, Karolinska Institutet POB 4064, SE-141 04 Huddinge, Sweden; tove.hasselblad@ki.se (T.H.), anna-stigsdotter.johansson@stud.ki.se (A.S.J.), anna.johansson.3@stud.ki.se (A.J.), goran.dahllof@ki.se (G.D.); 2 Center for Pediatric Oral Health Research, SE-171 77 Stockholm, Sweden

**Keywords:** congenital disorder, dental enamel, psychosocial stress, thematic analysis, qualitative study

## Abstract

Amelogenesis imperfecta (AI) is a hereditary developmental disorder affecting the enamel of teeth. Affected patients present with tooth hypersensitivity, rapid tooth wear, or fractures of enamel as well as alterations in color and shape, all of which compromise esthetic appearance and masticatory function. Chronic conditions in childhood severely impact the whole family, affecting normal family routines and/or increasing the family’s financial burden. The aim of this study was to explore experiences and the impact on daily life of being a parent to a child with severe forms of amelogenesis imperfecta. Parents of children and adolescents with AI participated in an interview with a psychologist. The transcribed interviews were analyzed using thematic analysis. The parents talked about several concerns about having a child with AI. Four main themes emerged from the interviews: Feelings associated with passing on a hereditary disorder, knowledge decreases stress, unfamiliarity with the diagnosis, and psychosocial stress. In these main categories we identified several subthemes. Feelings associated with passing on a hereditary disorder included the subtheme of guilt/shame; knowledge decreases stress included knowledge about diagnosis in the family and support from dental health care professionals; Unfamiliarity with diagnosis included missed diagnosis, fear of not getting correct treatment, and insufficient pain control; finally, the subtheme Psychosocial stress included fear of child being bullied and emergency dental visits. The findings show that parents of children with severe amelogenesis imperfecta report similar experiences as do parents of children with other chronic and rare diseases.

## 1. Introduction

Amelogenesis imperfecta (AI) is a hereditary developmental disorder affecting the enamel of teeth. The mode of inheritance is either autosomal dominant, autosomal recessive, or X-linked. Mutations in at least six genes have been shown to cause nonsyndromic AI [1]. The prevalence reported is between 1 in 700 to 1 in 14,000 depending on the population studied [2]. The severity of enamel disturbances in AI varies; the most affected patients present with tooth hypersensitivity, rapid tooth wear, or fractures of enamel as well as alterations in color and shape, all of which compromise esthetic appearance and masticatory function.

Current guidelines for restorative treatment in young children and adolescents suggest covering the surface with direct composite resin or composite resin veneers until adulthood and recommend stainless steel crowns for first permanent molars [3]. Pousette-Lundgren et al. [4] have shown that longevity of composite resin restorations is significantly lower in patients with AI and that patients require frequent replacements of fillings.

AI causes considerable distress in affected patients [5]. A qualitative study of adolescents with AI [6] reported severe pain, feelings of embarrassment, and dealing with dental staff that lack knowledge and understanding of their condition. Young individuals with AI also report decreased oral health-related quality of life [7].

Chronic conditions in childhood severely impact the whole family, affecting the normal family routines during the child’s hospitalization and/or increasing the family’s financial burden [8]. Parents also report increased stress as well as psychological and social difficulties [9]. Moreover, parents must sometimes accept the lack of an effective cure and must become involved in decisions about the treatment plan. Parents of children with osteogenesis imperfecta referred to their child’s pain as a highly stressful situation and reported feeling sad, anxious or incompetent, because pain is a permanent threat to their child’s well-being and they feel unable to avoid it [10].

AI is a hereditary disease. It has no cure, and is associated with fractures of teeth, pain, loss of restorations, frequent emergency visits, and decreased oral health-related quality of life, factors which all can contribute to increased stress in the family. The aim of this study was to explore experiences and impact on daily life of being a parent to a child with severe forms of amelogenesis imperfecta.

## 2. Materials and Methods

### 2.1. Recruitment of Participants

The study focuses on the experiences of parents of adolescents and young adults living with severe forms of AI who had received early crown therapy. Parents of children and adolescents treated in the public dental service in Dalarna, Sweden, and at the specialist pediatric dentistry clinic in Falun, whose children and adolescents had previously participated in a study on their experiences [6], were contacted by phone and asked to participate in an interview with a psychologist not involved in the treatment of these children. In-depth interviews were conducted during 2015, in places not in connection to dental clinics, and were audio-recorded. Nine parents were contacted and all agreed to participate. One parent cancelled the interview on the same day, due to the work situation. These parents were selected since they were parents of children with the most severe forms of AI who had been subjected to extensive dental treatment starting between 9 and 18 years of age. Some of the parents also had younger children with or without diagnosis. All patients came from middle-class families in small-town areas and all parents were employed. They were also selected because they lived close to the interview sites.

### 2.2. Interviews

The interviews, one-to-one, on one occasion followed a topic guide consisting of open-ended questions related to the experience of being a parent to a child diagnosed with severe AI who had undergone early prosthetic crown therapy [11]. The interviewer (TH), a psychologist with experience of treating patients with dental fear and anxiety, let the parent take the lead and asked follow up questions only when necessary or when the discussion lapsed. The interviews took between 10 and 65 minutes. All interviews were transcribed by two dental students in verbatim and controlled by three of the authors: TH, GPL, and GD. Pseudonyms are used for all the participants in this study.

### 2.3. Thematic Analysis

The transcribed interviews were analyzed using thematic analysis as described by Braun and Clark [12]. Four of the parents read the transcribed interviews for data source triangulation. They all agreed that the transcribed data corresponded to the recorded interviews. One parent stated “I stand for every word I have said”. All co-authors, one clinical psychologist, two specialists in pediatric dentistry, and two dental students independently analyzed the data. All themes were derived from the transcribed data. We then compared the results of these independent analyses and combined them over the course of several meetings until agreement of the themes and subthemes was reached.

We followed the six steps proposed by Braun and Clarke [12]. First, we familiarized ourselves with the data by listening to the recorded interview and reading and re-reading the transcribed interviews several times. After sorting the data, we searched for potential themes and created a mind-map with possible themes. For a theme to be valid, it had to satisfactorily answer the question “What is this expression an example of?” [12]. After identifying a set of candidate themes, we revised the themes and considered the validity of individual themes in relation to the data set. The analyzing process implied a movement back and forth between data and ideas of what would illuminate them. Three authors (ASJ, AJ, GPL) together tested how the themes fitted with further data in a scrutinizing and iterative process. If no consent was achieved around the themes and sub-themes, the discussion involved GD and TH and a consensus was achieved. Then we defined and redefined the themes. Two colleagues (a psychologist and a pediatric dentist) reviewed the final analysis and confirmed the results. After this the report was able to be written. The transcribed interviews are in Swedish and can be made available to researchers on request to the corresponding author. The goal of reporting these interviews in publications was explicit throughout and was included in the processes of research ethics approval and participant consent.

This study followed the Declaration of Helsinki guidelines and was approved by the Regional Ethics Review Board in Uppsala (Daybook 2014/481).

## 3. Results

### 3.1. Participants

In total, eight parents were interviewed. All were parent to at least one child with severe AI. Five parents had been diagnosed with AI since childhood, one was diagnosed as a result of their child being diagnosed, and two parents did not have AI (Table 1).

### 3.2. Experience of Being a Parent to a Child with Severe AI

The parents talked about several concerns about having a child with AI. The concerns were personal reflections as well as concerns about knowledge and communication resulting in environmental impact on the family.

**Figure 1:**
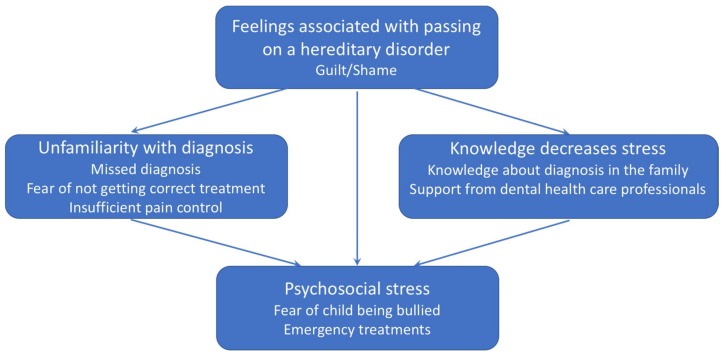
Themes and subthemes explaining the experience of being a parent to a child with severe amelogenesis imperfect.

Four main themes emerged from the interviews with parents of children with severe forms of AI: Feelings associated with passing on a hereditary disorder, Knowledge decreases stress, Unfamiliarity with the diagnosis, and Psychosocial stress. In these main categories we identified several subthemes. Feelings associated with passing on a hereditary disorder included the subtheme of guilt/shame; Knowledge decreases stress included knowledge about diagnosis in the family and support from dental health care professionals; Unfamiliarity with diagnosis included missed diagnosis, fear of not getting correct treatment, and insufficient pain control; finally, the theme of Psychosocial stress included fear of child being bullied and emergency dental visits (Figure 1). Regarding the relation between the themes, unfamiliarity with the diagnosis increases and knowledge decreases psychosocial stress. Denial of problems from dental professionals also adds guilt and shame as well as frustration when not being able to explain the child’s problems.

### 3.3. Feelings Associated with Passing on a Hereditary Disorder

#### Guilt/Shame

Inheritance was a common topic in the interviews and most parents reported feelings of guilt and shame in passing on a disease to one’s child.

*Now that he got the same as me, it was quite scary*

(John)

*This has been something very difficult for my husband who also has the disease… that he has passed this on to his children, he finds this very difficult, he blames himself for it, of course…he says he shouldn’t have had children, because you don’t pass on bad genes, and in a way, I can understand his way of thinking… he feels it’s hard to have given something so bad to his children, something that they will live with for the rest of their lives.*

(Ann-Sofie)

There were also children blaming their parents for passing on the disease.

*My children have been angry with me saying ’why did I get your damn teeth?’*

(Monica)

Feelings of guilt, shame, and passing on something that is not good was also mentioned in relation to future grandchildren. Some parents were worried that their grandchildren would get AI.

*I’m already worried about his children, he has not had any children yet but…you can’t really tell him not to have children just because of teeth…*

(John)

*My husband said that he sometimes wished that you could genetically manipulate the children so that they would not get the disease.*

(Monica)

Some parents had thoughts about genetic testing but none of the parents stated that it would affect their reproductive decisions.

### 3.4. Knowledge Decreases Stress

#### 3.4.1. Knowledge about Diagnosis in the Family

Another aspect of AI being hereditary disorder was expressed as finding strength from own experiences of having AI and being treated for AI or from having relatives with AI.

*I knew [about having AI]…thanks to knowing that there were so many of my relatives having it*

(Sandra)

*I knew there was something better [treatment], since I’ve experienced it myself.*

(Anita)

*Now that we know and if Sara were to have a child, she will know where to look for help and so on. That’s nice.*

(Jessica)

When someone has the disease in the family, the children can see the treatment outcome for their parents, older siblings or other relatives.

#### 3.4.2. Support from Dental Health Care Professionals

Meeting dental professionals with proper knowledge decreases stress for the parents. Parents explain how relieving it was to be treated with respect and knowledge from the dentists and to have their problems taken seriously.

*It was a young dentist, a girl, she had probably seen a lot in her schooling I can assume and she was curious. And she said straight away have you sought specialist care for this? I was like, finally someone reacts to this.*

(Christine)

A wish brought up by many parents was to have an information leaflet about the disease to avoid having to explain over and over again.

*When I got a new boss, and she just ’You go to the dentist so often with your daughter, do you really have to be away for a whole day? You really don’t want to see how she looks after a dental appointment, it doesn’t work to leave her at school in that state’. It would feel much better if you could give a leaflet that describes the disease and what it means for the patient. A lot of dental visits and a lot of problems.*

(Ann-Sofie)

### 3.5. Unfamiliarity with Diagnosis

The parents had extensive experience of dental treatment themselves and had very strong feelings regarding the treatment they or their children received and how they were met by dental health care. These experiences include missed diagnosis, fear of not getting correct treatment, and insufficient pain control.

#### 3.5.1. Missed Diagnosis

AI is a rare and not generally known disease even among dental health care professionals. Parents report that they have been met with ignorance, from both general dental practitioners and the general public. There is a frustration of not being understood.

*Everyone knows what being lactose intolerant is or having celiac disease. If you are allergic to dogs everyone knows what it is, but if you say that you have AI people just go “okay so what is that?” I mean, no one has any idea of what it is.*

(Ann-Sofie)

Even at the dentist, where parents expect to be met with knowledge and their children receive good care, this has always been the case. Instead, many have experienced that they have been poorly treated and accused of causing their children’s problems themselves. Even after receiving a diagnosis the dental staff did not always take the time to read about it in the records.

*I’ve experienced very little understanding and very little knowledge. Even though we are a small group, I think that if they are able to find out if there is a diagnosis in the records, they should be able to find out what it means.*

(Monica)

#### 3.5.2. Fear of Not Getting the Correct Treatment

Some parents have been aware of their own diagnosis, one had it confirmed after their child was diagnosed and some parents did not have AI. Regardless, most parents felt as if they had to fight for their children to get proper dental care. In some instances, parents felt as if it had been them, and not the dentist, who had noticed or brought up that something was wrong with their children’s teeth, often to find that the dentist did not agree.

*I was a bit annoyed, I can tell you, when they didn’t take it seriously, especially when I had told them … they did see how … how it looked … that can’t have looked normal in the eyes of a dentist either.*

(Sandra)

In general, parents felt that they had to take more than usual responsibility to make sure that their children were given the best possible treatment.

*You should probably stand up for yourself as a parent if you notice that something is wrong, yeah but like in her case that you probably shouldn’t count on that the dentist, refer you instead you have to stand up for yourself and if you suspect these things, say that we want a specialist to look at this.*

(Jessica)

*He is studying in Uppsala so I have to try to find a dentist for him there so he can go and check his teeth.*

(Sandra)

#### 3.5.3. Insufficient Pain Control

Parents had to push for their children to have more local anesthesia when being treated in general dentistry since the children still were in pain when a normal dose had been given.

*We had to explain about pain and the pain threshold to the dentists, that maybe it’s not enough with one anesthetic injection when they have to treat a tooth.*

(Monica)

The parents themselves thought that the dental staff regarded them as quite demanding parents and not so easy to deal with.

*They probably thought we were demanding parents, that we were overprotective regarding our children. That we… were kind of troublesome. I felt that way.*

(Monica)

### 3.6. Psychosocial Stress

As a result of living with a child with AI, parents experienced psychosocial stress, including the subthemes Fear of child being bullied and Emergency dental visits.

#### 3.6.1. Fear of Child Being Bullied

Many parents themselves pointed out that their children’s teeth are not so esthetically pleasing.

*Ugly teeth, sensitive teeth, broken teeth, fractures, cavities/decays and above all, so terribly ugly.*

(Annika)

Parents reported that their children had been bullied or teased due to esthetic problems. Some parents experienced this themselves as children. In addition to this, many parents said that their children did not want to smile or laugh without covering their teeth with their hands or lips.

*He was really teased so he has never smiled in a photo because he was teased in school. They asked if he didn’t brush his teeth and other mean things.*

(Monica)

*You have been teased […] both myself and my children.*

(Annika)

*I threatened the principal of the school to make complaint to the police regarding the bullying she was exposed to, it was not only the teeth, it was everything. It was such a relief when she finished school.*

(Sandra)

Having a different appearance and in addition to that being teased as a result was a stress factor.

#### 3.6.2. Emergency Dental Visits

Teeth with a tendency to fracture and failed restorations resulted in many emergency dental visits for the children and their parents. This creates stress in the family system because it takes time, unplanned time, and they also worried about pain associated with the visit.

*Yeah but well it’s been so very much, very much with those teeth, because sometimes they fall apart and she is in really bad pain and then we like have to drop work and go straight to the dentist, if we get an appointment.*

(Ann-Sofie)

*The teeth have fractures just about any time and when the children were small it was like panic every time, they called from kindergarten and school when they’ve been at things so you just have to go to the dentist with like half a front tooth.*

(Anita)

Even when traveling, the parents thought about where to go if something serious happened.

*If you travel and the teeth fracture… when Emma was younger, we had to go to the dentist time and time again. It was like if you were in another city it was like, is there an on-call dentist here?*

(Annika)

## 4. Discussion

The results of this qualitative interview study show that being a parent to a child with severe forms of amelogenesis imperfecta is associated feelings of guilt for having passed on a hereditary disease, stress associated with all aspects of dental care, and also a feeling of extra responsibility for the child’s well-being.

Although AI is not classified as a rare disorder in Sweden, it is still unknown to the general public. If knowledge about rare medical disorder is limited in society, knowledge about rare dental disorders is even more lacking. Since rare diseases also may vary with time and geographical area, general dentists may not regularly meet patients with AI or have experience of the particular challenges associated with treating patients with AI.

Most parents report feelings of shame and guilt for passing on a genetic disorder. The feeling of guilt is based in the experience of stigma that is associated with having a genetic disorder. In a study of hypohidrotic ectodermal dysplasia (XHED) [13], mothers of affected males reported feeling of guilt for passing on a genetic disorder and concerns about the stigma experienced by their affected sons. Similar to this study, trans-generational guilt was experienced by grandmothers passing on the disease to her sons and grandsons. In contrast to the situation with XHED, none of the parents reported that AI had any influence on their decision to have children, but some parents mentioned guilt and even worries about the next generation having children with AI.

For parents of children with AI it is of importance that they are met with respect and knowledge about their child’s condition from dental health professionals. In this study parents reported that they had been met with ignorance and even unwillingness to meet the special requirements necessary to make a proper diagnosis and treatment plan or to provide effective pain control during restorative treatment. This situation is similar as reported by Trulsson et al. [14], where families with children with rare disorders such as Rett and Angelman syndromes found it important that medical caregivers and other professionals around the child and the family had knowledge about the child’s disability. It was also perceived as important that professionals knew the physical consequences of the disability and how to treat the child. A study of parents of children with chronic physical disorders [15] found that staff underestimated parents’ emotional distress and need for information during admission of their chronically ill child.

In studies of parents of children with disabilities the importance of a diagnosis is central. Parents of children with a known diagnosis report better psychosocial well-being than parents of children with disabilities but with an unknown or uncertain diagnosis [16]. In this study, parents expressed the wish for an information leaflet they could give employers or teachers that can explain the disease and the need for frequent visits to dental health care and also the risk for acute events. For parents, understanding from the workplace and from schoolteachers is essential.

Parents of children with medical conditions or disabilities often report having to struggle to get what they think is the best for their children in terms of care and support [17]. Several parents reported that their children were not given adequate pain control during dental treatment. This is not uncommon in dentistry, since dentists are reported to underuse local anesthesia, analgesia, and sedation, particularly for preschool children [18]. In a recent study of dental treatment in 2363 children, Ghanei et al. [19] found that one-third of all treatment sessions were experienced as painful or causing discomfort. Many dentists describe a stressful working environment with financial constraints for child dental health care. In this environment children with rare conditions and more complex treatment needs may suffer. A holistic view, interprofessional collaboration, and a tolerant work environment are required to increase quality of dental health care for children with rare conditions [20].

Parents in this study reported that they themselves and their children had been bullied due to the unaesthetic appearance of their teeth. This is a common concern of parents with children with different forms of disabilities. Clarke [13] reported that mothers of boys with XHED worried about their sons being bullied or socially excluded during their school years. Teenagers with cleft lip and palate also experience teasing, bullying, and stares [21]. To varying degrees, they felt constricted in engaging in the community as they worried what others thought about their visible difference. We have previously shown that early crown therapy in teenage years in patients with AI results in a normalization of daily life with, self-assurance, and being able to act normally in relationships [6].

Parents of children with AI reported experiencing frequent emergency situation with fractures of teeth, failed restorations, pain, and difficulties accessing dental care as well as the stress involved in having to leave work to go with their children to the dentist. The longevity of dental restorations in children with AI is significantly shorter compared to a control group [4]. This means that the number of dental visits per year is also significantly higher in AI patients. Patients with AI also report high levels of tooth sensitivity requiring addition measures [11] which may not always be available in emergency situations. In patients with osteogenesis imperfecta, a rare bone disorder associated with increased bone fragility, fractures, pain, and emergency visits were considered highly stressful by the parents. The unpredictability and uncontrollable possibility of a new fracture contributed to parental stress as well as the consequent inability to protect the child [10].

In the interviews, the parents showed their frustration not to be listened to and the lack of a permanent long-lasting treatment. We have previously shown that ceramic crown therapy is possible with excellent long-lasting results without adverse events [11]. We have also shown an increase in oral health-related quality of life after crown therapy. The treatment that was carried out in adolescents, in some cases starting as early as 9 years of age [7].

In this study we used a one-to-one interview between parent and psychologist. In a previous study of children with AI using this design [6] adolescents reported more impacts on daily life compared to a focus group design [22]. Sensitive topics might be more likely to be discussed in a one-to one interview than in a focus group. In an individual interview it is also easier to explore an issue in depth and detail [23]. The interviews were undertaken by a psychologist working in a specialist pediatric dentistry setting with cognitive behavioral therapy for dental phobia. This is a strength of this study since all the contextual factors of patients with AI are known.

In conclusion, our findings show that amelogenesis imperfecta impacts families in much the same way as other chronic and rare diseases, with feelings of guilt and shame with respect to the transmission of a hereditary disease, difficulties in dealing with healthcare professionals, burden of emergency treatments, and uncertainty about the future. Situations of insufficient pain control during dental treatment are stressful. It is obvious that dental health care to a greater extent needs to increase participation of parents in dental health care, consulting parents as experts in aspects of care of their children as well as finding time to adjust the dental treatment to the specific needs of this patient group.

## Figures and Tables

**Table 1:** Characteristics of interviewed parents.

Name	Sex	Age	Parental AI	Number of Children	Children with AI
Annika	F	52	Yes	1F/1M	2/2
Monica	F	45	No **	1F/2M	2/3
Ann-Sofie	F	43	Yes	1F/2M	2/3
Sandra	F	50	Yes	1F/1M	2/2
Christine	F	48	No **	2F	1/2
Jessica	F	45	Yes *	1F/1M	1/2
John	M	50	Yes	1M	1/1
Anita	F	45	Yes	3F	3/3

AI: amelogenesis imperfect; F: female; M: male; * The mother was diagnosed after her daughter; ** Partner had AI.

## References

[1] Wright J.T., Torain M., Long K., Seow K., Crawford P., Aldred M.J., Hart P.S., Hart T.C. (2011). Amelogenesis imperfecta: Genotype-phenotype studies in 71 families. Cells Tissues Organs.

[2] Crawford P.J., Aldred M., Bloch-Zupan A. (2007). Amelogenesis imperfecta. Orphanet J. Rare Dis..

[3] McDonald S., Arkutu N., Malik K., Gadhia K., McKaig S. (2012). Managing the paediatric patient with amelogenesis imperfecta. Br. Dent. J..

[4] Pousette Lundgren G., Dahllöf G. (2014). Outcome of restorative treatment in young patients with amelogenesis imperfecta. a cross-sectional, retrospective study. J. Dent..

[5] Coffield K.D., Phillips C., Brady M., Roberts M.W., Strauss R.P., Wright J.T. (2005). The psychosocial impact of developmental dental defects in people with hereditary amelogenesis imperfecta. J. Am. Dent. Assoc..

[6] Lundgren G.P., Wickström A., Hasselblad T., Dahllöf G. (2016). Amelogenesis Imperfecta and Early Restorative Crown Therapy: An Interview Study with Adolescents and Young Adults on Their Experiences. PLoS ONE.

[7] Pousette Lundgren G., Karsten A., Dahllöf G. (2015). Oral health-related quality of life before and after crown therapy in young patients with amelogenesis imperfecta. Health Qual. Life Outcomes.

[8] Dogba M.J., Bedos C., Durigova M., Montpetit K., Wong T., Glorieux F.H., Rauch F. (2013). The impact of severe osteogenesis imperfecta on the lives of young patients and their parents—A qualitative analysis. BMC Pediatr..

[9] Cousino M.K., Hazen R.A. (2013). Parenting stress among caregivers of children with chronic illness: A systematic review. J. Pediatr. Psychol..

[10] Santos M.C.D., Pires A.F., Soares K., Barros L. (2018). Family experience with osteogenesis imperfecta type 1: The most distressing situations. Disabil. Rehabil..

[11] Pousette Lundgren G., Vestlund G.M., Dahllöf G. (2018). Crown therapy in young individuals with amelogenesis imperfecta: Long term follow-up of a randomized controlled trial. J. Dent..

[12] Braun V., Clarke V. (2006). Using thematic analysis in psychology. Qual. Res. Psychol..

[13] Clarke A. (2016). Anticipated stigma and blameless guilt: Mothers’ evaluation of life with the sex-linked disorder, hypohidrotic ectodermal dysplasia (XHED). Soc. Sci. Med..

[14] Trulsson U., Klingberg G. (2003). Living with a child with a severe orofacial handicap: Experiences from the perspectives of parents. Eur. J. Oral. Sci..

[15] Bradford R. (1991). Staff accuracy in predicting the concerns of parents of chronically ill children. Child Care Health Dev..

[16] Davies R., Davis B., Sibert J. (2003). Parents’ stories of sensitive and insensitive care by paediatricians in the time leading up to and including diagnostic disclosure of a life-limiting condition in their child. Child Care Health Dev..

[17] Graungaard A.H., Skov L. (2007). Why do we need a diagnosis? A qualitative study of parents’ experiences, coping and needs, when the newborn child is severely disabled. Child Care Health Dev..

[18] Wondimu B., Dahllöf G. (2005). Attitudes of Swedish dentists to pain and pain management during dental treatment of children and adolescents. Eur. J. Paediatr. Dent..

[19] Ghanei M., Arnrup K., Robertson A. (2018). Procedural pain in routine dental care for children: A part of the Swedish BITA study. Eur. Arch. Paediatr. Dent..

[20] Hallberg U., Strandmark M., Klingberg G. (2004). Dental health professionals’ treatment of children with disabilities: A qualitative study. Acta Odontol. Scand..

[21] Tiemens K., Nicholas D., Forrest C.R. (2013). Living with difference: Experiences of adolescent girls with cleft lip and palate. Cleft Palate Craniofac. J..

[22] Sneller J., Buchanan H., Parekh S. (2014). The impact of amelogenesis imperfecta and support needs of adolescents with AI and their parents: An exploratory study. Int. J. Paediatr. Dent..

[23] Patton M. (2015). Qualitative Research & Evaluation Methods.

